# Knowledge, practices and influencing factors defining unhealthy food behavior among adolescents in India: a scoping review

**DOI:** 10.3389/fpsyg.2023.1161319

**Published:** 2023-06-08

**Authors:** Susangita Jena, Jayashree Parida, Arpita Panda, Swati Sukalyani Behera, Abinash Pradhan, Prasanna Kumar Patra, Sanghamitra Pati, Harpreet Kaur, Subhendu Kumar Acharya

**Affiliations:** ^1^Regional Medical Research Centre (ICMR), Bhubaneswar, Odisha, India; ^2^Department of Anthropology, Utkal University, Bhubaneswar, Odisha, India; ^3^Division of ICMR, Division of Epidemiology and Communicable Diseases (ECD-Tribal Health), New Delhi, India

**Keywords:** unhealthy food, adolescents, knowledge, practices, influencing factors, India

## Abstract

Unhealthy food behaviors are the major contributing factors to the rising burden of non-communicable diseases (NCDs) among adolescents in India. Knowledge and practices related to unhealthy eating are significant factors influencing adolescents’ food behavior. In this scoping review, we aim to map evidence and identify gaps on knowledge, practices, and the influencing factors associated with unhealthy food behavior among Indian adolescents by examining the existing literature. Arksey and O’Malley’s scoping review framework and the Joanna Briggs Institute Reviewers’ manual were used for this review. After the screening, 33 articles were identified according to the inclusion criteria. Data extraction was performed according to the study objectives, followed by narrative summarization. The studies included a total of 20,566 adolescents. Most studies reported insufficient knowledge about healthy food choices among adolescents. Diet intake patterns among the adolescents showed a lower amount of fruits and vegetables and an increase in fried items, sugar-sweetened beverages, packaged food, and fast food in both sexes, with a broader association with peer influence (21.2%), parental unhealthy food behavior (15.1%), place of residence (6.06%), emotional status (6.06%), and mass media exposure (18.1%). The scoping review highlights the need for targeted interventions aimed at improving the knowledge and practices of Indian adolescents by promoting healthy food choices and sensitizing them about the risk of non-communicable diseases. The analysis of evidence around adolescent dietary behavior in India shows a monotonous, limited, and narrow range in scope, indicating the extended need for research.

## Introduction

Adolescence is the transition period between childhood and adulthood, ranging from 10 to 19 years, where adolescents experience rapid biological, cognitive, physiological, social, and emotional development (World Health Organization, 2022a). Adolescence is a vulnerable phase from the nutritional developmental prospect, and individuals at this age easily get attracted to unhealthy food behavior (Demory-Luce and Motil, 2014). In this context, unhealthy foods may be considered first prepared, rapidly available, and highly processed food, mostly with fewer nutrient values (Kaushik et al., 2011). A global Burden of Diseases (2019) study among 195 countries between the year of 1990–2017 reported that an unhealthy diet and lousy food consumption with nutritional deficiencies caused around 11 million deaths, which is much higher than the death caused by excessive tobacco consumption (Afshin et al., 2019). A United Nations Children’s Fund (UNICEF) report shows that in low and middle-income countries, about 42% of school-going adolescents consume carbonated sugary soft drinks at least once a day, and 46% eat fast food at least once a week. The statistics rise to 62 and 49%, among adolescents in high-income countries (UNICEF, 2019).

India has the world’s largest adolescent population (253 million; United Nations International Children’s Fund, 2022). In India, available evidence reported unhealthy food behavior among adolescents in numerous contexts. Studies have shown that adolescents due to inadequate knowledge about the involved health hazards, continue to consume unhealthy foods (Sharma, 2013; Shanthini and Hubballi, 2021), some other evidence reported the continuance of consumption of unhealthy food even after acquiring the necessary knowledge and awareness on the associated health hazards (Joseph et al., 2015; Sharma and Pokhrel, 2016). Gender plays a vital role in the dietary behavior of adolescents. In this context, male adolescents have been reported to have such behavior more than their female counterparts, particularly older male adolescents (Bhushan et al., 2017; Kuila et al., 2017). The quantity of food intake decreases among girls from early to late adolescence and boys prefer meat and fast food significantly and more frequently than girls (Askovic and Kirchengast, 2012).

Changing food habits is a part of human development; it reflects the transition in the cognitive level regarding making individual choices. Adolescence is marked with struggles for autonomy, intimacy, and friendships while handling peer exposure, which can all influence their eating habits (Fudge, 2013). In Indian studies, findings have examined a significant association of unhealthy food behavior with peer group influence, taste, place of residence, gender, and attractive advertisement (Joseph et al., 2015; Veena et al., 2018). Here it may be mentioned that an unhealthy diet contributes to several chronic diseases, including cardiovascular disease, cancer, type-2 diabetes, and other conditions associated with obesity among adolescents (World Health Organization, 2022b). It is crucial to note that a healthy diet throughout adolescence is essential since unhealthful eating habits during this period of life can develop into a long-term risk factor in adulthood (Lassi et al., 2017).

With the present exploratory analyses, it was found that, in India, there are bottlenecks in studies covering various aspects of unhealthy food behavior, particularly among adolescents. So, the current scoping review intends to give a comprehensive status of evidence on different selected areas of unhealthy food behavior among Indian adolescents, including junk food, fast food, and packaged food. The present synthesis of evidence and gap analysis will be valuable for future research on adolescent dietary behaviors. The objectives of the current scoping review were to evaluate the evidence around the status of knowledge and practices of adolescents towards unhealthy food in India and to explore the status of research on major factors associated with unhealthy food behavior among Indian adolescents.

## Methods and analysis

The present scoping review described the evidence around knowledge, practices, and influencing factors defining unhealthy food behavior among Indian adolescents. For this review, we followed five-stage of Arksey and O'Malley’s (2005) and Joanna Briggs Institute Reviewers’ Manual methodological frameworks, including (i) identification of research question; (ii) identification of relevant study; (iii) study selection; (iv) data charting; (v) collecting, summarizing, and reporting the result (Arksey and O'Malley, 2005; Peters et al., 2015).

### Identifying relevant studies

The framing of research questions, search strategy, and eligibility criteria was conducted based on the PCC (population, concept, and context) strategy (Peters et al., 2015).

1. What is the status of evidence around the knowledge and practices of Indian adolescents toward unhealthy food?
2. What literature available on influencing factors of unhealthy food practices among adolescents in India?

### Search strategy

The combination of MeSH terms like “unhealthy food”; “fast food”; “junk food”; “packaged food”; “processed food”; “preserved food”; “prevalence”; “knowledge”; “attitude”; “practices”; “risk factor”; “factor associate”; “adolescents”; “teenager”; “youth”; “India” from different online databases such as PubMed, Google Scholar, Google, EMBASE and SCOPUS database were used to identify relevant articles for this scoping review. Search terms were combined using Boolean operators (AND, OR, NOT). Relevant grey literatures were also considered for this review. The references of all the articles were also searched to find out additional articles. Supplementary File 2 (List of search databases) gives the search strategy used for the PubMed online database.

### Eligibility criteria

#### Inclusion Criteria

- Population: The adolescent group aged between 10 and 19 years was included.
- Concept: Literature with a specific focus on knowledge, practices, influencing factors, and risk factors of unhealthy food behavior among adolescents was identified. By knowledge, we mean knowledge regarding poor or unhealthy food and its harmful effect on human health and body. By practice, we mean the various eating and drinking practices around fast/processed food items.
- Context: The present analysis addressed unhealthy food behavior among Indian adolescents.
- Language of published literature: All the studies were published in English.
- Review period: The present review included studies conducted between the years 2000 to 2021.

#### Exclusion criteria

- Studies undertaken before 2000 and considered populations other than India were excluded.
- Studies targeting individuals below 10 years and above 19 years were excluded.
- Papers with no original data were excluded to avoid duplication of data.

### Study procedure and selection of the studies

Defined inclusion and exclusion criteria were followed to select the relevant literature. Duplicate articles were removed through crosschecking, and the original papers were evaluated and excluded based on the titles and abstracts of the articles. Based on the eligibility criteria, the two reviewers assessed the full text of the remaining papers (SJ and AP). If any disagreement arose, the third reviewer (JP) was consulted. All the stages of selecting the relevant studies were presented in the flow diagram as prescribed in Preferred Reporting Items for Systemic Review and Meta-analysis Scoping Reviews (PRISMA-ScR; Tricco et al., 2018; Supplementary File 3).

### Charting the data

The data from the selected literature were extracted manually by the reviewers (SJ and AP) and presented in a summary table using the MS Excel spreadsheet. Publication details included the author’s information, year of publication, area of study, sample technique, region, study settings, sample size, age group, gender, knowledge, practices, and influencing factors associated with unhealthy food behavior (Supplementary File 1).

### Collating, summarizing, and reporting the results

In the final step, the status of existing evidence on knowledge, practices, and influencing factors of unhealthy food behavior among Indian adolescents was collected to be categorized into different themes and summarized the results. The retrieved data were coded by identifying the major concepts and themes linked to unhealthy dietary behavior and its influencing factors. This technique involved labeling sections of data with descriptive codes that reflect the meaning of the text; in the following stage, we arranged similar codes together to uncover broad themes. Once the themes were identified, they were analyzed to better understand the knowledge, practices, and influencing factors related to unhealthy food behavior among Indian adolescents. The results of the included studies were compiled into a report to identify gaps for further research.

## Results

**Figure 1:**
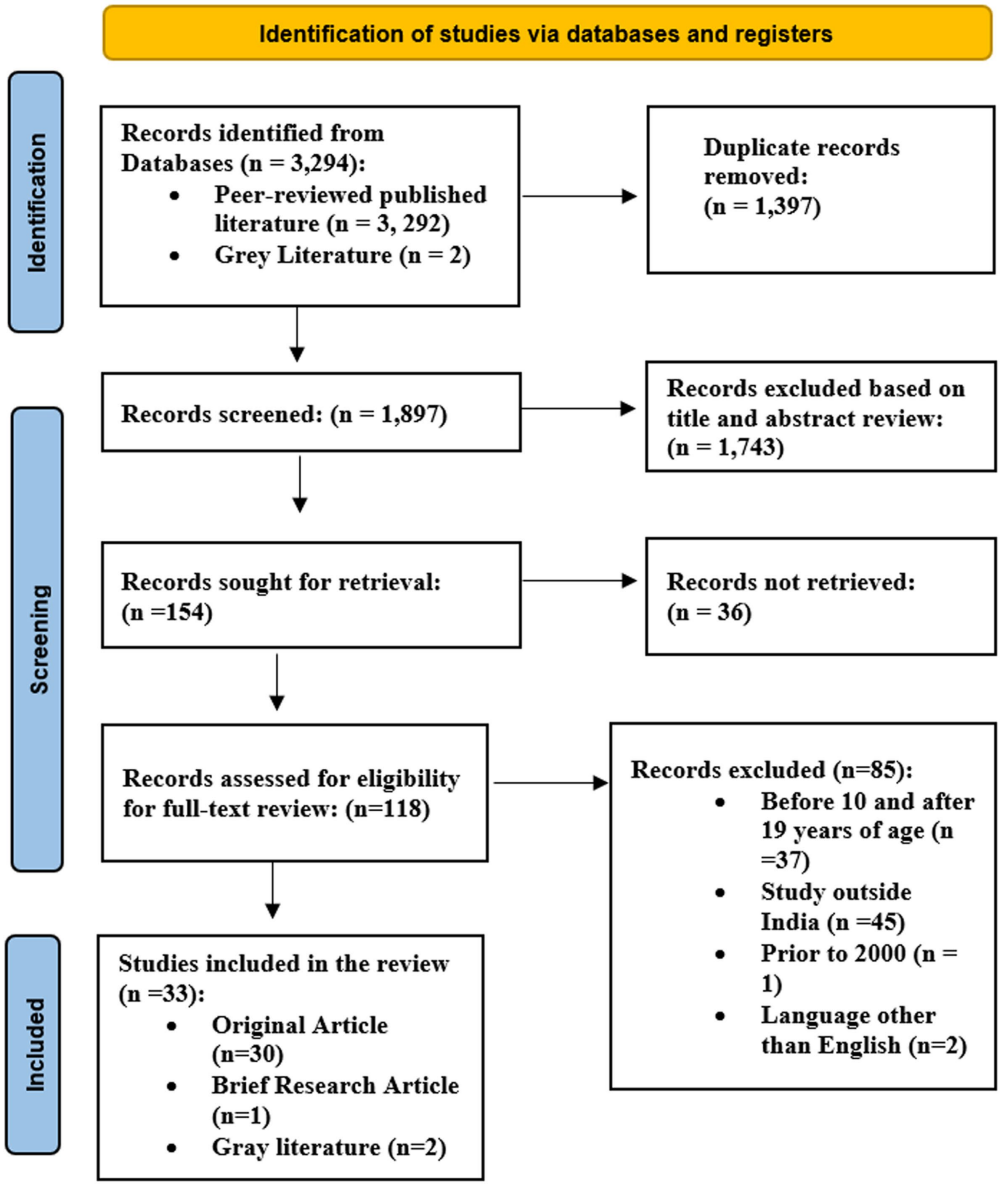
PRISMA flow chart (literature search result).

Figure 1 represents the PRISMA flow chart explaining the literature review process. In total, 3,294 searches were located from different electronic databases, including grey literature like websites of agencies, academic institutions, and technical bodies. Following the removal of 1,397 duplicate records, a total of 1,897 records were reviewed, with 1,743 records being excluded due to title and abstract screening. Among the 154 retrievals records, 118 full-text records were suitable for analysis, and 33 of them were included in the research based on inclusion and exclusion criteria (Figure 1).

**Figure 2:**
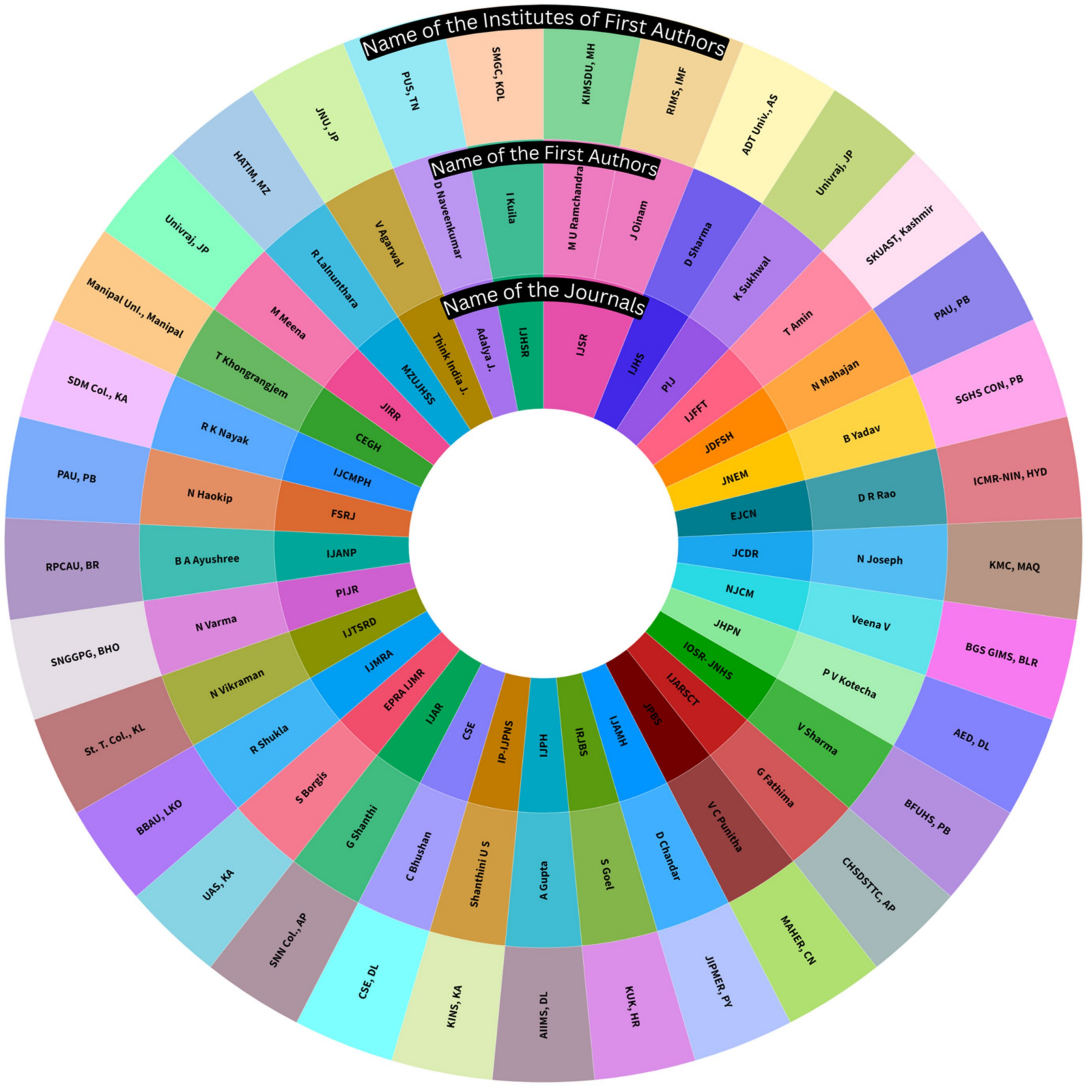
Bibliography of the selected studies presenting pattern of research continuance by researchers, institutional representation, and publishing journals on adolescent unhealthy food behavior from India.

The bibliographic analysis in Figure 2 shows the overall pattern of research pursued around adolescents’ unhealthy food behavior by individual researchers in India, institutions involving these studies, and the journals selected for publishing the included studies (Supplementary File 4). The bibliometric analysis indicates that publications are sporadic and there is no consistent pursuit of studies on unhealthy food consumption among Indian adolescents.

A summary table (Table 1) for the selected 33 studies in the review represented information on the author, year of publication, area of study, sample techniques, region, type of study, study settings, sample size, age group, gender, method, knowledge, and practices of unhealthy food behavior, and factor associated/ influencing factor. Seventeen of the total 33 studies were conducted on adolescent junk food behavior while another 12 were on fast food behavior. The summary table represents brief information regarding the characteristics of the included study. Most of the eligible evidence was published in the last 10 years and all included publications reported quantitative outcomes (Table 1). Overall, the pattern of studies undertaken showed an inconsistent trend of research during the years 2000–2021.

**Table 1:** Characteristics of included studies.

Demographic variable	*N*	% Out of 33 publications included
*Year of publication*		
2000–2010	1	3.03%
2011–2021	32	96.96%
*Source of article*		
Original Article	30	90.90%
Brief Research Article	1	3.03%
Gray literature Case Report (*n* = 1) Government Survey Report (*n* = 1)	2	6.06%
*Regional distribution*		
Northern zone	10	30.30%
North-eastern zone	3	9.09%
Central zone	2	6.06%
Eastern zone	2	6.06%
Western zone	2	6.06%
Southern zone	13	39.39%
Multicentric	1	3.03%
*Type of data*		
Quantitative	31	93.93%
Qualitative or mixed method	2	6.06%
*Community*		
Multicentric	1	3.03%
Rural	2	6.06%
Urban	3	9.09%
Rural and urban	4	12.12%
Urban and semi urban	2	6.06%
Not known	21	63.63%
*Settings*		
Community-based	3	9.09%
School-based	30	90.90%
*Gender*		
Girls	8	24.24%
Boys	2	6.06%
Both	23	69.69%
*Study indicators of unhealthy food*		
Knowledge and perception	18	54.54%
Unhealthy food practices	26	78.78%
Influencing factors/correlates	15	45.45%

### Geographical distribution of the evidence

**Figure 3:**
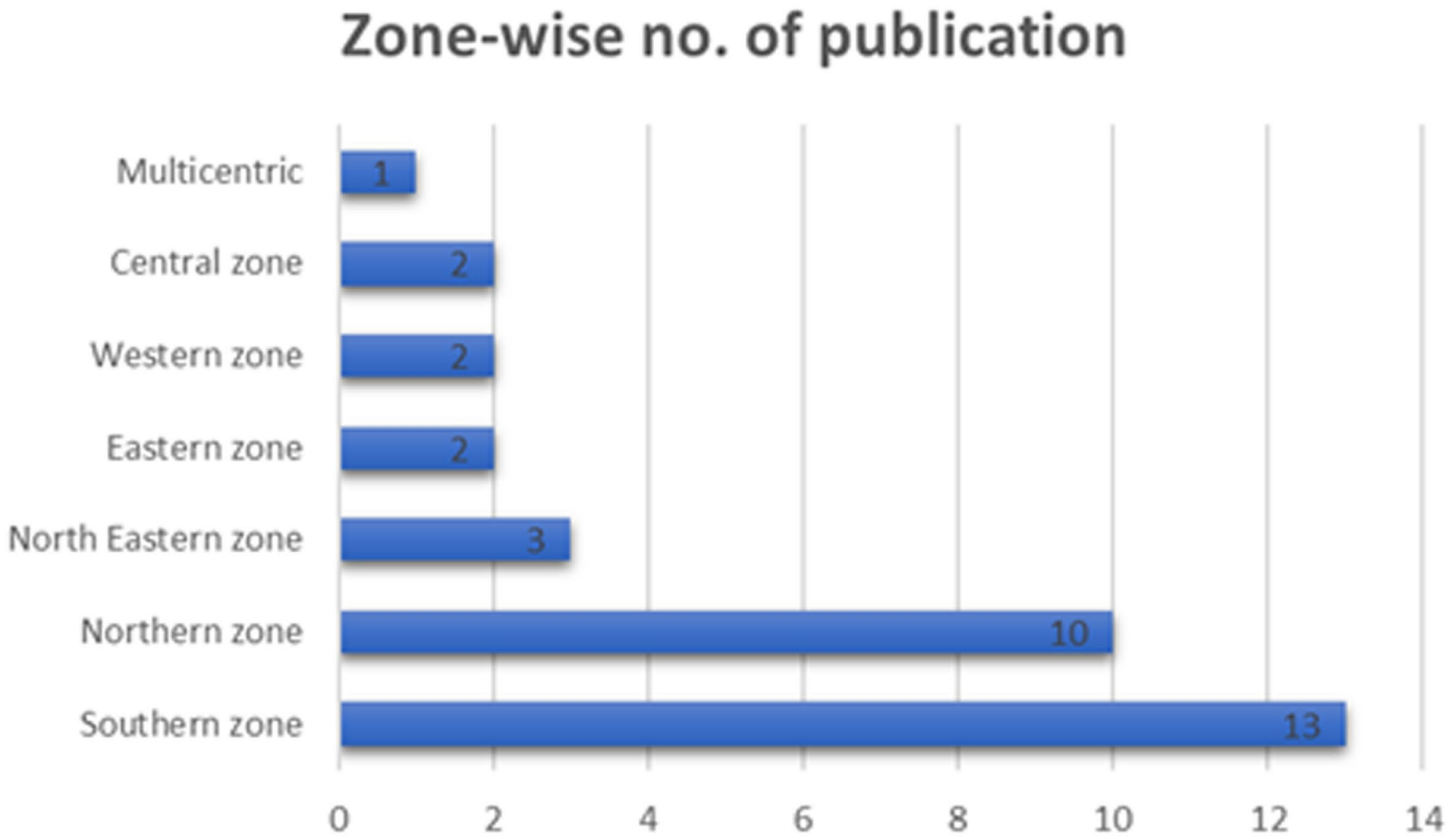
Zone-wise number of publications in India.

Out of the 33 studies, most publications (*n* = 13) were conducted in the southern region of India. One large-scale study that covered 25 states/UT of India (Bhushan et al., 2017). School-based/institute-based studies were reported in the highest (*n* = 30/33) number of papers (Figure 3).

### Target population

Sex-stratified analysis was provided in 23 studies. Eight records reported only data on girls, whereas two documents contained only boys-related information. The total sample size in the selected 33 studies was 20,566, with an average of 623 adolescents. In terms of participants’ age, four records focused exclusively on the age range of 10–19 years (Kotecha et al., 2013; Kuila et al., 2017; Shukla et al., 2017; Chandar et al., 2020), and the maximum number (*n* = 19) of studies conducted among late adolescent age group (15–19 years; Table 1).

### Study indicators for unhealthy food practices

Of the total included studies, 18 (54.54%) papers covered the area of knowledge and awareness about unhealthy food behavior among Indian adolescents where studies discuss adolescents’ knowledge regarding the consequences and characteristics of unhealthy food behavior (Table 2).

**Table 2:** List of selected study indicators explaining the unhealthy food practices.

Study indicators	Number of publications
*Knowledge and perception of unhealthy food*	
Knowledge about the consequences of unhealthy food	15 (45.45%)
Knowledge about the characteristics of unhealthy food	6 (18.18%)
Source of knowledge	2 (6.06%)
Gender differences in knowledge and perception of unhealthy food	10 (30.30%)
*Unhealthy food practices*	
Type of food practice	21 (63.63%)
Frequency of unhealthy food consumption	8 (24.24%)
Sharing partner and place of consumption	3 (9.09%)
*Factors influencing unhealthy food choices*	
Peer influence	7 (21.21%)
Parental unhealthy food behavior	5 (15.15%)
Carving for taste	7 (21.21%)
Place of residence	2 (6.06%)
Mass media exposure	6 (18.18%)
Emotional status	2 (6.06%)

#### Knowledge and perception of unhealthy food

##### Knowledge about the consequences of unhealthy food

Fifteen studies (45.45%) covered the status of knowledge concerning the effects of unhealthy food among adolescents; effects include causing over-weight, obesity, hypertension, high cholesterol, intestinal cancer, cardiovascular disease, dental caries, etc. due to consumption of unhealthy food (Sharma, 2013; Joseph et al., 2015; Ramchandra et al., 2015; Sharma and Pokhrel, 2016; Bhushan et al., 2017; Vikrama and Nitha, 2017; Khongrangjem et al., 2018; Naveenkumar and Parameshwari, 2019; Shanthi et al., 2019; Yadav and Kaur, 2019; Borgis et al., 2020; Lalnunthara and Kumar, 2020; Nayak, 2020; Ayushree and Tarai, 2021; Shanthini and Hubballi, 2021). One study reported the health consciousness of adolescents while eating unhealthy food (Lalnunthara and Kumar, 2020).

##### Knowledge about the characteristics of unhealthy food

Six studies (18.18%) evaluated the status of knowledge regarding the aspects of unhealthy food containing high sugar and salt, increasing calories, lacking nutritionalw value, rising cholesterol levels, causing weight gain, and containing harmful colors, chemicals, high in saturated fat, repetition of the same frying oil, food preservatives, etc. along with the knowledge regarding the consequences of consuming that (Kotecha et al., 2013; Sharma and Pokhrel, 2016; Amin et al., 2017; Vikrama and Nitha, 2017; Veena et al., 2018; Borgis et al., 2020; Ayushree and Tarai, 2021). In two studies, the participating adolescents opined that unhealthy food causes ill effects on the human body (Joseph et al., 2015; Vikrama and Nitha, 2017).

##### Source of knowledge

A significant section (93.93%) of the included studies did not explain the source of knowledge regarding particular food behavior and patterns of unhealthy food behavior among the participating adolescents. Only two papers explored these aspects and reported that mass media, friends, parents, and health professionals were the primary sources of information on hazardous and unhealthy food behavior (Vikrama and Nitha, 2017; Sukhwal et al., 2020).

##### Gender differences in knowledge and perception of unhealthy food

Only two studies reported on unhealthy food consumption among male adolescents (Mahajan et al. (2012); Joseph et al., 2015), with Mahajan et al., 2012 reporting that consumption was higher among urban male adolescents than among rural male adolescents (Mahajan et al., 2012). Female consumption was documented in eight studies (Raghunatha Rao et al., 2007; Goel et al., 2013; Meena and Varma, 2014; Shukla et al., 2017; Vikrama and Nitha, 2017; Chandar et al., 2020; Sukhwal et al., 2020; Fathima and Moni Meghana, 2021). Only two studies reported gender differences in knowledge about the health hazards of unhealthy food (Vikrama and Nitha, 2017; Naveenkumar and Parameshwari, 2019). Boys are more aware of the harmful effects of chemicals used in food than girls.

#### Unhealthy food practices

Unhealthy food contains low-essential nutrients that our body requires. Twenty-six (78.78%) of the included studies discussed unhealthy food practices among Indian adolescents (Table 2).

##### Type of food practices

Twenty-one (63.63%) articles of the study reported on the variety of unhealthy food consumed by adolescents; Chocolate, packaged food, bakery food, fried food, Italian food, Chinese food, and carbonated drinks are the most preferred food among adolescents (Raghunatha Rao et al., 2007; Mahajan et al., 2012; Goel et al., 2013; Kotecha et al., 2013; Meena and Varma, 2014; Joseph et al., 2015; Punitha et al., 2015; Oinam et al., 2016; Sharma and Pokhrel, 2016; Varma and Chaturvedi, 2016; Amin et al., 2017; Bhushan et al., 2017; Gupta et al., 2018; Khongrangjem et al., 2018; Veena et al., 2018; Agarwal et al., 2019; Naveenkumar and Parameshwari, 2019; Borgis et al., 2020; Nayak, 2020; Sukhwal et al., 2020; Ayushree and Tarai, 2021; Fathima and Moni Meghana, 2021). Two studies reported frequent consumption of locally made unhealthy food or food with low nutrients and high saturated fats like Aloo Tikki, bhelpuri, samosa, chole kulcha, chole bhatura, Bikaneri bhujia, Gol Gappa among adolescents of semi-urban areas (Mahajan et al., 2012; Sukhwal et al., 2020).

From the above results, eight papers discussed food items in detail under the junk food category; they defined junk food as high in calories, low in nutritional value and mainly containing high sugar and fat. Eleven studies represented food item details under the fast-food category, where they defined fast food that was prepared and served quickly, often high in calories, fat and salt. In one study, Bhushan et al. (2017) described detail about the consumption of packaged food among adolescents, where he defined packaged food as food that was pre-packaged for convenience. Upon further detailed analysis, the unhealthy food practices in a few of the studies were contextually classified under various subcategories as follows (Table 3);

- *Unhealthy food under the Indian Traditional food category* (Mahajan et al., 2012; Sukhwal et al., 2020)—Alootikki, Bhelpuri, Samosa, Chole kolache, Chole bhature, Golgappe/Panipuri, Chat, Namkeen etc.
- *Unhealthy food practices under the locally available Italian food categories* (Mahajan et al., 2012; Goel et al., 2013)—Pizza, Pasta, Macroni, French Fries, Sandwiches, etc.
- *Unhealthy food practices under the locally available Chinese food categories* (Goel et al., 2013; Khongrangjem et al., 2018; Sukhwal et al., 2020)—Maggie, Manchurian, Noodles, Spring rolls, Momo, Fried Chicken, etc.
- *Sweet dish* (Goel et al., 2013)—Chocolate, Ice cream
- *Beverage* (Goel et al., 2013; Amin et al., 2017; Bhushan et al., 2017; Gupta et al., 2018)—Carbonated drinks, Cold drinks, and soft drinks; a study by Amin et al., 2017 also revealed that adolescents highly preferred Carbonated drinks along with fast food than any other drink/Juice. And adolescents preferring this kind of drink were mentioned in the above 15 included studies.
- *Bakery Products* (Gupta et al., 2018; Naveenkumar and Parameshwari, 2019; Nayak, 2020)—Pastry, Cake, Patties, Creemroll, Puffs, Doughnuts etc.

**Table 3:** List of unhealthy food practices defined in the selected studies.

Sl No.	Studies and years	Regional representation	Age group/gender	Reported unhealthy food items
1	Sukhwal et al. (2020)	Northern India	15–17, Female	Traditional food (Aloo Tikki, bhelpuri, samosa, chole kulcha, chole bhatura, Bikaneri bhujia, Gol Gappa), Chocolate, Chips, Chinese snacks
2	Amin et al. (2017)		16–19, Male and Female	Carbonated drink
3	Mahajan et al. (2012)		16–18, Male	Fried food, Chinese food, Traditional fast food (aloo tikki, bhelpuri, samosa, fryams, pav Bhaji, chhole kulche, Chhole bhature, cream roll, veg and paneer pakora), Italian food, Soft drinks
4	Goel et al. (2013)		16–18, Female	Bakery items, Italian food (Pizza, Pasta, Macaroni), Chinese (Maggie, Manchurian, Noodles, Spring rolls), Sweet dishes (Chocolate, Ice cream), Fried food (Bread pakoda, chat, kachori, samosa, potato chips, tikki), Beverages (carbonated drink, coffee)
5	Gupta et al. (2018)		12–18, Male and Female	Chips, Chocolate, Bakery products (pastry, cream roll, patties, soft drinks), Sweetened beverages (sweet juices, squash), Sweets, Ice cream, Samosa
6	Haokip and Sonika (2016)		16–19, Male and Female	Burger, Manchurian, Noodles, Pastry, Pizza, Samosa
7	Meena and Varma (2014)		14–16, female	Ice cream, Cold drinks, Indian sweets, Wafer/Chips, Cold coffee, Chocolate, Pizza, Pastry, Kachori/Samosa, Maggie, Biscuit, Namkeen, Indian sweets
8	Joseph et al. (2015)	Southern India	12–15, Male	Pizza, Burger, Samosa, Chocolate
9	Veena et al. (2018)		16–18, Male and Female	Potato chips, Deep fried snacks, Ice-cream, Pizza, Burger, Noodles, Soft drinks
10	Fathima and Moni Meghana (2021)		17–19, Female	Potato chips, Soft drinks, Pizza, Ice-cream, Panipuri, Noodles, Fried Rice, Samosa, Puffs
11	Punitha et al. (2015)		13–19, Male and Female	Carbonated drink
12	Borgis et al. (2020)		13–16, Male and Female	Chips, nankeens, sweets, toffees, candies
13	Nayak (2020)		16–19, Male and Female	Chocolate, Soft drinks, Chips, Bakery product, Noodles, Chats
14	Khongrangjem et al. (2018)		15–19, Male and Female	Pastries, Pizza, French Fries, Cheese Items, Chinese Food, Soft drinks, Cold coffee, Sweetened fruit drink
15	Naveenkumar and Parameshwari (2019)		14–19, Male and Female	Fast food (pizza, burger, fries, noodles), Snacks, Soft drink, Candies, Bakery food (puffs, pastry, cake, doughnuts)
16	Haokip and Sonika (2016)	Western India	10–19, Male and Female	Burger, Manchurian, Noodles, Pastry, Pizza, Samosa.
17	Varma and Chaturvedi (2016)	Central India	13–19, Male and Female	Italian food, Burger, Chinese food, Fried food
18	Ayushree and Tarai (2021)	Eastern India	15–19, Male and Female	Soft drinks, French fries, Fried Chicken, Pizza Hut, Dominoes, Chips, Chocolate, Burger
19	Kuila et al. (2017)		10–19, Male and Female	Eggroll, Noodles, Samosa
20	Oinam et al. (2016)	North-Eastern India	16–19, Male and Female	Noodles, Momo, Fried Chicken, Burger, Pakoras/Bora, Pizza, Samosa
21	Bhushan et al. (2017)	Multicentric India	10–17, Male and Female	Packaged food, Instant noodles, Sugar-Sweetened Beverages (SSB), Salted packaged food, sweetly packaged food (chocolate, ice cream), sweet beverage (carbonated beverage, packaged juice-based, packaged milk based)

##### Frequency of unhealthy food consumption

Eighteen studies (54.54%) reported the frequency of unhealthy food consumption habits observed daily to occasional. (Raghunatha Rao et al., 2007; Mahajan et al., 2012; Goel et al., 2013; Kotecha et al., 2013; Joseph et al., 2015; Punitha et al., 2015; Haokip and Sonika, 2016; Varma and Chaturvedi, 2016; Amin et al., 2017; Bhushan et al., 2017; Kuila et al., 2017; Khongrangjem et al., 2018; Veena et al., 2018; Agarwal et al., 2019; Naveenkumar and Parameshwari, 2019; Borgis et al., 2020; Chandar et al., 2020; Ayushree and Tarai, 2021). Out of these studies, two studies explored the study setup and defined the frequency of food consumption, where they found out that the frequency of unhealthy food consumption was high among urban adolescents (Mahajan et al., 2012; Sukhwal et al., 2020). A study by Veena et al. (2018) showed that adolescents were highly habituated to junk food, mainly as a snack item (Veena et al., 2018). A similar study by Sharma and Pokhrel (2016) reported that unhealthy food consumption is one of the favorite leisure-time activities among adolescents (Sharma and Pokhrel, 2016). Two studies said that adolescents preferred unhealthy food over usual meals (Joseph et al., 2015; Amin et al., 2017). Another report by Sharma and Pokhrel (2016) reported that most adolescents do not carry lunch boxes to school; hence they eat fast food during their lunchtime and replace one of their usual meals with fast food (Sharma and Pokhrel, 2016). Adolescents also reported that the carve for diverse tastes of foods was the driving force for preferring fast food outside. They believed that fast or processed foods available outside are tasty (Joseph et al., 2015; Sharma and Pokhrel, 2016).

##### Sharing partner and place of consumption

Three studies (9.09%) reported that adolescents usually continued their unhealthy food practice (fast food, junk food, preserved food, packaged food) with friends, family, and relatives (Oinam et al., 2016; Veena et al., 2018; Naveenkumar and Parameshwari, 2019), whereas evidence revealed that fast food stalls, restaurants, hostels, school canteens, and other eateries in nearby school area were the primary access points of such unhealthy food (Joseph et al., 2015; Punitha et al., 2015; Haokip and Sonika, 2016; Bhushan et al., 2017; Chandar et al., 2020).

##### Gender differences in unhealthy food practices

Among the total included studies, 23 were conducted among both male and female participants, with three mentioning that the unhealthy food intake rate was higher among male participants than female participants. (Varma and Chaturvedi, 2016; Bhushan et al., 2017; Ayushree and Tarai, 2021). According to Bhushan et al. (2017), male adolescents’ consumption of SSBs (Sugar-sweetened drinks) and salted packaged food was higher than female adolescents’ consumption of sweet packaged food (Bhushan et al., 2017). Another research conducted by Varma and Chaturvedi (2016) found that intake of unhealthy food more than twice a week was common among male adolescents; unhealthy food consumption as a snack was more common among male adolescents than females (Ayushree and Tarai, 2021). According to Agarwal et al. (2019), females enjoy eating fast food more than boys, and the primary reason is carving for taste. According to a study by Vikrama and Nitha (2017); the emotional state was an essential factor that influenced female adolescents to consume more unhealthy food, and most of them were unaware of the harmful impacts of chemicals in this food (Vikrama and Nitha, 2017). Teenage females suffer from skin infections, stomach aches, dysmenorrhea, and other symptoms due to consuming these unhealthy foods (Fathima and Moni Meghana, 2021).

#### Factors influencing unhealthy food choices

Many studies explored the major influencing factors of unhealthy food practices among adolescents. About 15 (45.45%) of the studies reported a correlation between unhealthy food behavior among adolescents (Table 2). Below, we have discussed these observed major factors.

##### Peer influence

Seven studies (21.21%) examined the association between peer group influence with unhealthy food practices (Goel et al., 2013; Kotecha et al., 2013; Oinam et al., 2016; Bhushan et al., 2017; Vikrama and Nitha, 2017; Naveenkumar and Parameshwari, 2019; Nayak, 2020). Of these, two papers indicated that popularity and peer group recommendations were major influencing factors for the choice of fast or processed food (Kotecha et al., 2013; Chandar et al., 2020). However, none of the studies clearly came up with findings that explained the role of peer pressure in unhealthy food practices among adolescents.

##### Parental unhealthy food behavior

We evaluated the effects of parental food behavior on children around unhealthy food during the present study. Five studies (15.15%) explored the relationship between parental unhealthy food behavior with adolescents’ food practices (Goel et al., 2013; Joseph et al., 2015; Naveenkumar and Parameshwari, 2019; Ayushree and Tarai, 2021). One study reported a significant influence of relatives’ food behavior on the children (Naveenkumar and Parameshwari, 2019). However, one study reported a family history of unhealthy food consumption (Veena et al., 2018) by multivariate analysis, which showed a significant association between age group, family history of obesity, and junk food eating frequency.

##### Carving for taste

Seven research studies (21.21%) investigated the link between adolescents’ desire for diverse teste and unhealthy food consumption (Oinam et al., 2016; Sharma and Pokhrel, 2016; Khongrangjem et al., 2018; Veena et al., 2018; Agarwal et al., 2019; Chandar et al., 2020; Nayak, 2020). Three studies cited the variety and the eating alternatives with less effort as major influential factors for preferring fast or processed foods. (Haokip and Sonika, 2016; Khongrangjem et al., 2018; Chandar et al., 2020). However, two studies reported that regular intake of home-cooked meals and the wish to change the taste of food regularly led to more unhealthy food consumption, which in several cases become a habit (Joseph et al., 2015; Veena et al., 2018).

##### Place of residence

Two studies reported that unhealthy food behavior is strongly associated with a place of residence like a hostel (Sharma and Pokhrel, 2016; Veena et al., 2018). One study assessed the association between economic status with unhealthy food behavior (Sharma and Pokhrel, 2016), reporting a positive association whereas two studies reported that the frequency of unhealthy food consumption was high among urban adolescents (Mahajan et al., 2012; Sukhwal et al., 2020).

##### Mass media exposure

Research stated that exposure to mass media, either print or electronic is the primary factor driving the rise in unhealthy eating habits. Six studies described how these eye-catching advertisements tempt adolescents to increase unhealthy food consumption (Joseph et al., 2015; Sharma and Pokhrel, 2016; Khongrangjem et al., 2018; Chandar et al., 2020; Lalnunthara and Kumar, 2020; Sukhwal et al., 2020).

##### Emotional status

The association between emotional states and unhealthy eating behavior was examined in two research studies. (Sharma and Pokhrel, 2016; Varma and Chaturvedi, 2016). A study by Varma and Chaturvedi (2016) reported that adolescents enjoyed eating unhealthy food (junk food, fast food, packaged food) thus, they converted it into regular meals (Varma and Chaturvedi, 2016).

## Discussion

This review identified 33 empirical studies published between 2000 and 2021 presenting details on the characteristics of unhealthy food intake behavior among Indian adolescents. The results posited patterns of evidence and status of adolescents’ knowledge and practices about unhealthy food behavior. Evidence regarding the consumption pattern of junk food, fast food, packaged food, ultra-preserved food, and carbonated beverages was also identified. We analyzed the selected cross-sectional studies to assess the knowledge and awareness about unhealthy food practices in different settings in India. This review identified limited knowledge and awareness regarding the consequences of unhealthy food among adolescents. In contrast, they knew some of the disadvantages from mass media, health professionals, and family members (Yadav and Kaur, 2019). The findings of the selected studies indicate that unhealthy food carries high saturated fat, high sugar content, high cholesterol, and low fiber, leading to several health conditions like weight gain and other chronic diseases (Kotecha et al., 2013; Joseph et al., 2015). As reported by another study, more information regarding the associated health hazards is insufficient for adolescents to stop eating unhealthy food consumption (Amin et al., 2017). Twelve studies reported intake patterns of unhealthy food and showed that adolescents having such a habit consumed junk food 3–4 times per week; Chocolate, instant noodles, fried food, bakery item, Chinese food, fast food, and carbonated drinks were the most preferred items in these studies. According to the Comprehensive National Nutrition Survey (CNNS) 2016–2018 reports, about 30% of adolescents consume fried food once a week (Comprehensive National Nutrition Survey: 2016–2018, 2019). The recent government report on adolescent nutrition in India found that almost 81 million boys and 63 million girls were either thin, short, anemic, overweight, or obese due to unhealthy food practices (Comprehensive National Nutrition Survey: 2016–2018, 2019). In India, a survey report by Bhushan et al. (2017) indicated that the packaged food consumption rate was high among late adolescent boys. Overall evidence suggested that the unhealthy consumption trend was higher among urban and late adolescents than among rural or early adolescents.

Gender is an important factor in defining food habits. A gender-based difference in unhealthy food habits was important in some of the selected studies. Studies highlighting gender differences related to unhealthy food behavior suggested that junk food and fast-food consumption were prevalent among all genders in Indian adolescents and at high frequency. Some studies reported a higher prevalence of junk food habits among adolescent males (Bhushan et al., 2017; Kuila et al., 2017; Ayushree and Tarai, 2021). On the other hand, a study from Tamil Nadu indicated an increased level of inadequate nutritional knowledge attached with increased affiliation to junk food among adolescent girls than boys (Naveenkumar and Parameshwari, 2019). Another study conducted in Guwahati, in Assam, found that girls were more likely to engage in emotional eating behavior (Sharma and Pokhrel, 2016) as a coping mechanism for stress, depression, and negative emotions. On the other hand, boys were more likely to engage in over-eating behaviors and consume large portions (Scaglioni et al., 2018). A study from Mizoram found that adolescent girls faced more health-related problems after eating unhealthy food than boys (Lalnunthara and Kumar, 2020). However, in an overall context, the status of research from a gender perspective was observed as insufficient.

Globalization directly impacts rapid or dynamic changes in food practices, with increased availability and affordability of cheap food in local markets (Black, 2016). Merchandises are engrossing individuals to influence their current food behavior through attractive advertisements and publicity in different settings (e.g., schools, colleges, and canteen; Smith et al., 2019). However, some available studies in the Indian scenario suggested that the consumption of unhealthy food was primarily associated with some distinct sociodemographic variables such as place of residence (Mahajan et al., 2012; Sharma and Pokhrel, 2016; Veena et al., 2018), parental occupation (Ayushree and Tarai, 2021), educational qualification (Rathi et al., 2017), family size (Kotecha et al., 2013), amount of pocket money given (Yadav and Kaur, 2019), and availability of food (Khongrangjem et al., 2018; Nayak, 2020), etc. The selected evidence also showed that emotional states like boredom, stress and depression were independently associated with unhealthy food practices (Sharma and Pokhrel, 2016). Similarly, consumption patterns were also reported to be governed by the availability of food, diversity in food types, and distance of fast-food outlets (outcome: skipping breakfast or lunch). Furthermore, good taste, attractive advertisements, diversity of food (variety of options), the appearance of a favorite star/actor/sports/comic/animated character in the package, and limited options during hunger were associated factors. The place of residence of adolescents was also found to have a great impact on unhealthy dietary behavior; those who are living in the hostel along with access to outside food were found to be more addicted to unhealthy food due to the reasons like poor quality of food served in the hostel, time management, etc. (Veena et al., 2018). The important effect of parents’ food behavior on children was demonstrated in a few more studies where it was observed that adolescents living with their parents also consume more unhealthy food due to parents’ unhealthy food habits. However, the ‘friends’ factor’ was observed to be the critical influencing factor encouraging unhealthy food consumption, where food behavior was mainly defined due to the influence of friends. There are recommendations that urbanization, growing fast-food outlets, the busy lifestyle, and fast service are other factors influencing unhealthy dietary behavior among the adolescent population.

This review draws attention to the paucity of research on several aspects of perceptions and awareness of Indian adolescents of harmful eating habits. Since many studies have focused on the issue among the late adolescent category, there is a significant gap in the early adolescent age range. It may here be mentioned that in a similar vein, none of the research studies have looked into the qualitative component, which is important for learning more about the study’s objectives. Institution-based studies made up a sizable portion of investigations indicating a lack of evidence around community-based factors and patterns among adolescents. As the studies mostly aimed at their participants from institutes like schools and colleges, there is almost a vacuum regarding the unhealthy dietary behavior of drop-out adolescents. Additionally, it was observed that fewer research studies have considered male adolescents in comparison to females during data collection. The role of gender in food preference was also biased and less explored in the available studies.

We also briefly explored the policy perspective in India. We found that considering the concerns around poor food behavior among adolescents, the Food Safety and Standards Authority of India (FSSAI) came out with ‘The Eat Right Movement’ and new food safety guidelines, These steps emphasized “banning the sale of pre-packaged/junk foods in school vicinity,” which are referred to as foods with high in Fat, Salt, and Sugar (FSS) to school children in school canteens/hostel kitchens/mess premises or within 50 meters of the school campus (Gupta et al., 2019). This act may partially minimize the scope of access for children/adolescents from unhealthy food habits.

The following were the indicators under the act:

- High fat, salt, and sugar (HFSS) cannot be sold in the school canteen, mess premises, or hostel kitchen or within 50 m of the school campus.
- School canteen and kitchen need to get FSSAI licenses to operate.
- Maintain hygiene under schedule 4 of the Food Safety and Standard Act.
- Regular inspection by municipality authorities and state administration.
- School authorities adopt comprehensive programs to promote the consumption of a safe and healthy diet among school children. (National Institute of Nutrition).

However, we broadly found limited policies addressing the issue of poor food behavior among children in India. We understand that it is a common impression in the country to lack any policy around healthy food behavior as obvious, where as high as > 40% of children suffer from childhood undernutrition. However, it is high time to understand that a significant proportion of children are at a concerning risk of getting obese and other morbidities with several chronic lifestyle diseases.

In the above context, we observed that the available evidence is incomprehensive and limited in finding while researches are inconsistent. By analyzing the authors/ corresponding authors, affiliated institutions, and the publishing journals, we observed no such consistency from the reporting research groups or scientists, and institutes in pursuing studies on adolescent unhealthy food behavior. Rather the studies are more incidental and sporadic (Figure 2). This circumstance highlights the critical need for more consistent and focused research in India’s adolescent nutrition and metabolic health. Addressing these research gaps will result in new, more inclusive, and complete research outputs in adolescent health, leading to the establishment of efficient policies for healthy food practices among adolescents.

### Strengths and limitations of the study

This scoping review is the first of such studies on knowledge, practices, and influencing factors defining unhealthy food practices among adolescents in India as per the electronically available information. This evidence-based study will enormously benefit for further research and future research policy. However, the study is limited on the basis of the selected language of evidence as we only focused the English language and electronically available databases.

## Conclusion

Unhealthy food behavior among Indian adolescents has become a significant concern in recent years, which is causing several non-communicable diseases and poor growth outcomes among them in short-term and later in adulthood. The finding indicates that various factors influence unhealthy food behavior among adolescents, including sociocultural, environmental, and individual factors, Additionally, inadequate knowledge about good nutrition and food choices also plays a crucial role in unhealthy food behaviors. Similarly, unhealthy food is defined differently in different regions in India, indicating a strong cultural and geographic variation; this implies a more inclusive definition of unhealthy food in Indian context. Most of the studies were replicative of previous studies in the context of their aims, objectives, methods and tools. We understand that there are enough opportunities for redefining the scopes contextually. Studies exploring adolescents’ evolving patterns of food and diet habits need to be undertaken to understand the problem properly. Qualitative exploration of various aspects of unhealthy food habits can bring in essential explanations to individual food behaviors that are leading to such harmful food practices.

Similarly, from a prevention perspective, a strong integrative partnership between various stakeholders, including policymakers, educators, parents, and adolescents, in various spaces like home, schools, public places, and utility facilities is required to create a supportive environment that encourages healthy eating habits. Furthermore, government policy around adolescent dietary and food practices needs to be modified and improved occasionally. Our analysis of available food policy in the country indicates a possible limitation on the fronts like implementation research and proper assessment which is causing major challenges to understand and addressing harmful food behavior among adolescents at home, schools, and other places.

## Recommendations

As highlighted, the selected studies presented limited scopes regarding factors associated with unhealthy food behaviors. So, any recommendations will not be sufficient as several aspects of adolescents’ unhealthy food behaviors are yet unexplored. Considering the findings and suggestions of the selected studies in the present review, a broader consensus of recommendations was developed, presented below.

- Proper implementation of government policies: It is imperative to implement the available government policies around preventing unhealthy diets and promoting a good diet in various spears of children’s lives.
- Bringing awareness: The government, schools, and healthcare experts ought to initiate awareness programs to inform adolescents about the value of a balanced diet and the adverse effects of poor eating habits.
- Nutrition education: Incorporating nutrition education into the school curriculum and health care settings can help adolescents establish good eating habits and make knowledgeable food choices.
- Peer influence: Adolescents’ poor food choices are frequently impacted by their peers; hence, interventions aimed at peer groups, such as social media campaigns, can be beneficial in encouraging healthy eating habits.
- Parental involvement: Because parents significantly influence their children’s food choices, boosting parental engagement in promoting healthy eating habits can positively impact their food choices.
- Food environment: Adolescent food behavior can be influenced by the availability and accessibility of unhealthy food in the food environment, such as fast-food outlets and convenience stores. Strict laws to control the availability and marketing of unhealthy food in the near-by food environment of schools can encourage healthy food choices.
- Physical activity: Physically active Adolescents tend to consume healthier foods, thus encouraging physical exercise can help them develop good eating habits.
- Research: Further research is necessary to discover the root causes that contribute to unhealthy food behavior among Indian adolescents. This can aid evidence-based interventions and policies promoting healthy eating habits among adolescents in India.

## Author contributions

SA: conceptualization and fund acquisition. SJ, ArP, JP, SB, and AbP: investigation. SJ, JP, and SA: methodology. SJ: writing—original draft preparation. SA, JP, PP, SP, and HK: writing—review and editing. All authors contributed to the article and approved the submitted version.

## Conflict of interest

The authors declare that the research was conducted in the absence of any commercial or financial relationships that could be construed as a potential conflict of interest.

## Publisher’s note

All claims expressed in this article are solely those of the authors and do not necessarily represent those of their affiliated organizations, or those of the publisher, the editors and the reviewers. Any product that may be evaluated in this article, or claim that may be made by its manufacturer, is not guaranteed or endorsed by the publisher.
