# Supplementary material for: Knowledge, practices and influencing factors defining unhealthy food behavior among adolescents in India: a scoping review
Source: Front Psychol. 2023 Jun 8;14:1161319. doi: 10.3389/fpsyg.2023.1161319 (PMC10285663; doi:10.3389/fpsyg.2023.1161319)
Supplement: Supplementary file 1 [file Data_Sheet_1.DOCX]

**Supplementary File 1**

**Data Extraction Table:**

| **SL NO.** | **AUTHOR**  **(YEAR)** | **PLACE OF STUDY;**  **STUDY DESIGN;** | **AGE GROUP; GENDER** | **UNHEALTHY FOOD PRACTICES** | **ATTITUDE KNOWLEDGE TOWORDS UNHEALTHY FOODS** | **RISK FACTOR/ FACTOR ASSOCIATE OF UNHEALTHY FOODS** |
| --- | --- | --- | --- | --- | --- | --- |
| 1 | Sharma and Pokhrel  (2016) | Guwahati, assam;  Cross-sectional | 19;  Male and Female | **Prevalence of consumption**  **Types of food preferred-**fast food, did not carry packed lunch | **Knowledge about consequences** | **influencing factor-** taste, convenience, Place of residence, attractive advertisement, emotional status, high socioeconomic status |
| 2 | Sukhwal and Verma  (2020) | Jaipur and Bhunas;  Cross-sectional | 15-17;  Female | **Prevalence of consumption-**high in urban  **Type of food preferred** = Traditional food (Aloo Tikki, bhelpuri, samosa, chole kulcha, chole bhatura, Bikaneri bhujia, Gol Gappa), Chocolate, Chips, Chinese snacks | NA | **influencing factor-** attractive advertisement |
| 3 | Amin et al.  (2017) | J & K;  Cross-sectional | 16-19;  Male and Female | **Type of food preferred=** carbonated drink  **Frequency of food consumption**  **Preference of fast foods over homemade meal** | **Knowledge about characteristics-** high sugar content of fast foods, high salt content of fast foods, fast foods have high saturated fat content, high cholesterol level, fast foods contain additives, low fiber content, not cooked under healthy conditions | NA |
| 4 | Mahajan et al.  (2011) | Punjab;  Cross-sectional | 16-18;  Male | **Frequency of consumption**  **Prevalence of consumption-**high in urban  **Type of food preferred=** Fried food, Chinese food, Traditional fast food (aloo tikki, bhelpuri, samosa, fryams, pav Bhaji, chhole kulche, Chhole bhature, cream roll, veg and paneer pakora), Italian food, Soft drinks | NA | NA |
| 5 | Yadav and Kaur  (2019) | Punjab;  Cross-sectional | 12-14;  Male and Female | NA | **knowledge about health consequences**  **source of knowledge regarding unhealthy food** | NA |
| 6 | Rao et al.  (2007) | Hyderabad;  Cross-sectional | 16-18;  Female | **Frequency of food consumption** | NA | NA |
| 7 | Joseph et al.  (2015) | Mangalore;  Cross-sectional | 12-15;  Male | **Prevalence of fast food**  **Frequency of consumption**  Preference for fast foods over the usual meal  **Type of food preferred=** Pizza, Burger, Samosa, Chocolate  fast food Preference for Presence of branded fast food, Preference of aerated drinks over fresh fruit juices | **knowledge of health consequences consumer-** tendency to cause weight gain, parental pressure to quit, waste of money  **Non-consumer-reason for harm full effect** contain harmful color agent, food preservative, unfresh food high-fat content, cause rapid weight gain, cause intestinal cancer, same frying oil use repeatedly  **Not aware of ill effects** need to control= 18.9%, no need to control=81.1% | **Influencing factors=** being bored with home food, Parental consumption  **Place of consumption** home, School premises, Fast food stall |
| 8 | Veena et al.  (2018) | Bangalore;  Cross-sectional | 16-18;  Male and Female | **Frequency of consumption- consume as evening snacks**  **Type of food preferred=** Potato chips, Deep fried snacks, Ice-cream, Pizza, Burger, Noodles, Soft drinks | **Knowledge about characteristics-** high calories food, make person overweight, junk food is not healthy, make person overweight | **influencing factors-** taste preference, easily available, convenience, lifestyle change, time constraint, attractive advertisement  **place of residence=** hosteler-increased eating after joining hostel, hostel food was not good, lack of time, junk food served in the hostel  **consume with whom-** friends, while watching tv |
| 9 | Kotecha et al.  (2013) | Baroda;  Cross-sectional | 10-19;  Male and Female | **Frequency of consumption**  **Type of food preferred=** packaged food, fried food, bakery food, Italian food, Chinese food, deep-fried snacks | **Knowledge about characteristics-** high in sugar, fat, and calories; bad for health, and lacking nutritional value | **influencing factor=**peer influence |
| 10 | Sharma  (2013) | Jalandhar, Punjab;  Cross-sectional | 15-17;  Male and Female | NA | **knowledge about health consequences** | **influencing factors=** availability of junk food in school =100% |
| 11 | Fathima & Meghana  (2021) | Elure, Andhra Pradesh;  Cross-sectional | 17-19;  Female | **Type of food preferred-** Potato chips, Soft drinks, Pizza, Ice-cream, Panipuri, Noodles, Fried Rice, Samosa, Puffs | NA | **risk factor=**skin infection, stomach ache, dysmenorrhoea, thyroid, anemia, sinus |
| 12 | Punitha et al.  (2015) | Chennai;  Cross-sectional | 13-19;  Male and Female | **Type of food preferred-**Junk foods, carbonated drink  **Frequency of consumption** | NA | **risk factor=** dental caries |
| 13 | Chandar et al.  (2018) | Puducherry;  Cross-sectional | 10-19;  Female | **Frequency of consumption** | NA | NA |
| 14 | Goel et al.  (2013) | Kurukshetra, Haryana;  Cross-sectional | 16-18;  Female | **Frequency of consumption**  **Type of food preferred** Bakery items, Italian food (Pizza, Pasta, Macaroni), Chinese (Maggie, Manchurian, Noodles, Spring rolls), Sweet dishes (Chocolate, Ice cream), Fried food (Bread pakoda, chat, kachori, samosa, potato chips, tikki), Beverages (carbonated drink, coffee) | NA | **Sources of purchasing junk food-**Home prepared, Any other  **risk factor=** over weight/obesity |
| 15 | Ramchandra et al.  (2015) | Karad, Maharashtra;  Cross-sectional | 17-20;  Male and Female | NA | **knowledge about health consequences** | **Sources of purchasing junk food**= hostel |
| 16 | Gupta et al.  (2018) | Kullu, Himachal Pradesh;  Cross-sectional | 12-18;  Male and Female | **Prevalence of food consumption**  **Type of food preferred=** Chips, Chocolate, Bakery products (pastry, cream roll, patties, soft drinks, Sweetened beverages (sweet juices, squash), Sweets, Ice cream, Samosa | NA | NA |
| 17 | Shanthini et al.  (2021) | Belagavi city, Karnataka;  Cross-sectional | 15-19;  Male and Female | NA | **knowledge about health consequences** | NA |
| 18 | Bhushan et al.  (2017) | 25 states & UT;  Cross-sectional | 10-17;  Male and Female | **Type of food preferred =** Packaged food, Instant noodles, Sugar-Sweetened Beverages (SSB), Salted packaged food, sweetly packaged food (chocolate, ice cream), sweet beverage (carbonated beverage, packaged juice-based, packaged milk based)    **Frequency of consumption** | **knowledge about health consequences-** not harmful to health, they add to health benefits | **influencing factors** Tasty, Attractive advertisement, Have limited options when hungry, Most friends have them, It is cool to have them, Favourite actor/sports star/comic character appears, Are usually consumed in family **Children eating or buying from schools or nearby areas (out of those consuming more than twice per week)**Carbonated beverages =62.2%, Packaged juice-based beverages=62.6%, Packaged milk-based beverages=48.8%, Chips Instant =64.5%, noodles =42.7% Chocolates=64.1%, Ice-cream=51.9% |
| 19 | Shanthi et al.  (2013) | Kasmira Nagar, Andhra Pradesh;  Cross-sectional | 16-20;  Male and Female | NA | **knowledge about health consequences** | NA |
| 20 | Borgis et al.  (2020) | Dharwad, Karnataka;  Cross-sectional | 13-16;  Male and Female | **Frequency of consumption**  **Type of food preferred=** Chips, nankeens, sweets, toffees, candies | **knowledge about health consequences**  **Knowledge about characteristics-** presence of harmful chemicals | **Influencing factors**- attractive advertisement |
| 21 | Shukla et al.  (2017) | Barabanki, Uttar Pradesh;  Cross-sectional | 10-19;  Female | **Prevalence of consumption**  **Frequency of consumption** | NA | NA |
| 22 | Vikraman  (2017) | Ernakulam, Kerala;  Cross-sectional | 17-19;  Male and Female | NA | **knowledge about health consequences**  **Knowledge about characteristics-** chemical present, unhealthy, eating on daily basis makes them eat more **source of knowledge regarding unhealthy food** | NA |
| 23 | Varma & Chaturvedi  (2016) | Bhopal;  Cross-sectional | 13-19;  Male and Female | **Type of food preferred=** Italian food, Burger, Chinese food, Fried food  **Frequency of consumption** | NA | NA |
| 24 | Ayushree & Tarai  (2021) | Odisha;  Cross-sectional | 15-19;  Male and Female | **Type of food preferred** Soft drinks, French fries, Fried Chicken, Pizza Hut, Dominoes, Chips, Chocolate, Burger  **Frequency of consumption as a meal**  **Frequency of consumption as snacks** | **knowledge about health consequences**  **Knowledge about characteristics-** unhealthy | **influencing factor-**Family member unhealthy food consumption |
| 25 | Haokip et al.  (2016) | Ludhiana;  Cross-sectional | 16-19;  Male and Female | **Frequency of consumption**  **Type of food preferred-** Burger, Manchurian, Noodles, Pastry, Pizza, Samosa.  **Ate junk food as an alternative to the main meal** | NA | **Place of consumption**  Students’ home canteen, College canteen, Roadside vendors, Fast food stall, Restaurants |
| 26 | Oinam et al.  (2016) | Imphal, Manipur;  Cross-sectional | 16-19;  Male and Female | **Prevalence of food intake**  **Type of food preferred** = Noodles, Momo, Fried Chicken, Burger, Pakoras/Bora, Pizza, Samosa  **Read nutrient information label before buying packaged food** | NA | **influencing factors** Taste, Convenience, Change in lifestyle  **Place of consumption**  Fast food center/restaurants/shop, School canteen, Home  **Usually have fast food (With whom**) Friends, Family, Alone |
| 27 | Nayak  (2020) | Karnataka;  Cross-sectional | 16-19;  Male and Female | **Type of food preferred** Chocolate, Soft drinks, Chips, Bakery product, Noodles, Chats | **knowledge about health consequences** | **influencing factors** taste, no other food option available, pleasure, easy availability, habit |
| 28 | Khongrangjem et al.  (2018) | Udupi, Karnataka;  Cross-sectional | 15-19;  Male and Female | **Frequency of food consumption** **Type of food preferred**  Pastries, Pizza, French Fries, Cheese Items, Chinese Food, Soft drinks, Cold coffee, Sweetened fruit drink | **knowledge of health consequences** | **influencing factors-**taste, attractive advertisements, diversity of fast-food Types, Convenience, availability of fast-food restaurants |
| 29 | Meena & Vermak  (2015) | Jaipur, Rajasthan;  Cross-sectional | 14-16;  Female | **Type of food preferred-**ice-cream, cold drinks, Indian sweets, wafer/chips, cold coffee, chocolate, pizza, pastry, kachori/samosa, Maggie, biscuit, namkeen | NA | NA |
| 30 | Lalnunthara & kumar  (2020) | Mizoram;  Cross-sectional | 16-19;  Male and Female | NA | **knowledge of health consequences** | **risk factor** health problem after eating fast food, instant food |
| 31 | Agarwal et al.  (2019) | Jaipur, Rajasthan;  Cross-sectional | 14-19;  Male and Female | **Frequency of food consumption** | NA | **influencing factors**  convenience, taste, price, variety of option |
| 32 | Naveenkumar & Parameshwari  (2019) | Tamil Nadu;  Cross-sectional | 14-19;  Male and Female | **Type of food preferred =** Fast food (pizza, burger, fries, noodles), Snacks, Soft drink, Candies, Bakery food (puffs, pastry, cake, doughnuts) | **knowledge of health consequences** | **Usually have fast food (With whom)** friends, family, relatives |
| 33 | Kuila et al.  (2017) | Medinipur, west Bengal;  Cross-sectional | 10-19;  Male and Female | **Frequency of consumption**  **Type of food preferred =** Eggroll, Noodles, Samosa | NA | NA |

**Supplementary File 2:**

**Search strategy MEDLINE (PubMed)**

Search conducted April 20, 2022, date range from January 2000 to December 2021

| **Search** | **Query** | **Records retrieved** |
| --- | --- | --- |
| #1 | ((("unhealthier"[All Fields] OR "unhealthiness"[All Fields] OR "unhealthy"[All Fields]) AND ("food"[MeSH Terms] OR "food"[All Fields])) OR ("fast foods"[MeSH Terms] OR ("fast"[All Fields] AND "foods"[All Fields]) OR "fast foods"[All Fields] OR ("fast"[All Fields] AND "food"[All Fields]) OR "fast food"[All Fields]) OR "junkfood"[All Fields] OR ("ultra"[All Fields] AND ("fast foods"[MeSH Terms] OR ("fast"[All Fields] AND "foods"[All Fields]) OR "fast foods"[All Fields] OR ("processed"[All Fields] AND "food"[All Fields]) OR "processed food"[All Fields])) OR (("drug packaging"[MeSH Terms] OR ("drug"[All Fields] AND "packaging"[All Fields]) OR "drug packaging"[All Fields] OR "package"[All Fields] OR "packages"[All Fields] OR "product packaging"[MeSH Terms] OR ("product"[All Fields] AND "packaging"[All Fields]) OR "product packaging"[All Fields] OR "packaged"[All Fields] OR "packaging"[All Fields] OR "packagings"[All Fields]) AND ("food"[MeSH Terms] OR "food"[All Fields])) OR (("carbohydrate s"[All Fields] OR "carbohydrated"[All Fields] OR "carbohydrates"[MeSH Terms] OR "carbohydrates"[All Fields] OR "carbohydrate"[All Fields]) AND ("drink"[All Fields] OR "drinking"[MeSH Terms] OR "drinking"[All Fields] OR "alcohol drinking"[MeSH Terms] OR ("alcohol"[All Fields] AND "drinking"[All Fields]) OR "alcohol drinking"[All Fields] OR "drinkings"[All Fields] OR "drinks"[All Fields]))) AND (2000:2021[pdat]) | 53,843 |
| #2 | ("adolescences"[All Fields] OR "adolescency"[All Fields] OR "adolescent"[MeSH Terms] OR "adolescent"[All Fields] OR "adolescence"[All Fields] OR "adolescents"[All Fields] OR "adolescent s"[All Fields] OR "adolescent"[MeSH Terms] OR "adolescent"[All Fields] OR "teenage"[All Fields] OR "teenager"[All Fields] OR "teenagers"[All Fields] OR "teenaged"[All Fields] OR "teenager s"[All Fields] OR "teenages"[All Fields] OR "adolescent"[MeSH Terms] OR "adolescent"[All Fields] OR "youth"[All Fields] OR "youths"[All Fields] OR "youth s"[All Fields]) AND (2000:2021[pdat]) | 1,414,621 |
| #3 | India: ("india"[MeSH Terms] OR "india"[All Fields] OR "india s"[All Fields] OR "indias"[All Fields]) AND (2000:2021[pdat]) | 586,740 |
| #4 | #1 AND #2 AND #3 | 120 |

**Supplementary File 3:**

**Preferred Reporting Items for Systematic reviews and Meta-Analyses extension for Scoping Reviews (PRISMA-ScR) Checklist**

| **SECTION** | **ITEM** | **PRISMA-ScR CHECKLIST ITEM** | **REPORTED ON PAGE #** |
| --- | --- | --- | --- |
| **TITLE** | | | |
| Title | 1 | Identify the report as a scoping review. | 1 |
| **ABSTRACT** | | | |
| Structured summary | 2 | Provide a structured summary that includes (as applicable): background, objectives, eligibility criteria, sources of evidence, charting methods, results, and conclusions that relate to the review questions and objectives. | 2 |
| **INTRODUCTION** | | | |
| Rationale | 3 | Describe the rationale for the review in the context of what is already known. Explain why the review questions/objectives lend themselves to a scoping review approach. | 3-4 |
| Objectives | 4 | Provide an explicit statement of the questions and objectives being addressed with reference to their key elements (e.g., population or participants, concepts, and context) or other relevant key elements used to conceptualize the review questions and/or objectives. | 4 |
| **METHODS** | | | |
| Protocol and registration | 5 | Indicate whether a review protocol exists; state if and where it can be accessed (e.g., a Web address); and if available, provide registration information, including the registration number. | N/A |
| Eligibility criteria | 6 | Specify characteristics of the sources of evidence used as eligibility criteria (e.g., years considered, language, and publication status), and provide a rationale. | 4-5 |
| Information sources | 7 | Describe all information sources in the search (e.g., databases with dates of coverage and contact with authors to identify additional sources), as well as the date the most recent search was executed. | 5 |
| Search | 8 | Present the full electronic search strategy for at least 1 database, including any limits used, such that it could be repeated. | 4 |
| Selection of sources of evidence | 9 | State the process for selecting sources of evidence (i.e., screening and eligibility) included in the scoping review. | 5 |
| Data charting process | 10 | Describe the methods of charting data from the included sources of evidence (e.g., calibrated forms or forms that have been tested by the team before their use, and whether data charting was done independently or in duplicate) and any processes for obtaining and confirming data from investigators. | 5 |
| Data items | 11 | List and define all variables for which data were sought and any assumptions and simplifications made. | 5 |
| Critical appraisal of individual sources of evidence | 12 | If done, provide a rationale for conducting a critical appraisal of included sources of evidence; describe the methods used and how this information was used in any data synthesis (if appropriate). | N/A |
| Synthesis of results | 13 | Describe the methods of handling and summarizing the data that were charted. | 5 |
| **RESULTS** | | | |
| Selection of sources of evidence | 14 | Give numbers of sources of evidence screened, assessed for eligibility, and included in the review, with reasons for exclusions at each stage, ideally using a flow diagram. | 6 |
| Characteristics of sources of evidence | 15 | For each source of evidence, present characteristics for which data were charted and provide the citations. | 6-7 |
| Critical appraisal within sources of evidence | 16 | If done, present data on critical appraisal of included sources of evidence (see item 12). | N/A |
| Results of individual sources of evidence | 17 | For each included source of evidence, present the relevant data that were charted that relate to the review questions and objectives. | Given in Supplementary file 1 |
| Synthesis of results | 18 | Summarize and/or present the charting results as they relate to the review questions and objectives. | 6-11 |
| **DISCUSSION** | | | |
| Summary of evidence | 19 | Summarize the main results (including an overview of concepts, themes, and types of evidence available), link to the review questions and objectives, and consider the relevance to key groups. | 11-14 |
| Limitations | 20 | Discuss the limitations of the scoping review process. | 14 |
| Conclusions | 21 | Provide a general interpretation of the results Provide a general interpretation of the results with respect to the review questions and objectives, as well as potential implications and/or next steps. questions and objectives, as well as potential implications and/or next stes. | 14 |
| **FUNDING** | | | |
| Funding | 22 | Describe sources of funding for the included sources of evidence, as well as sources of funding for the scoping review. Describe the role of the funders of the scoping review. | 16 |

**Supplementary File 4**:

List of journals and Institutes of 1^st^ Authors.

| **Sl no.** | **Authors Name** | **Journal Name** | **Journal Abbreviation** | **Institute Name** | **Institute Abbreviation** |
| --- | --- | --- | --- | --- | --- |
| 1 | D Sharma | International Journal of Home Science | IJHS | Asham Down Town University, Asham | ADT Univ., AS |
| 2 | K Sukhwal | The Pharma Innovation Journal | PIJ | University of Rajasthan, Jaipur | Univraj, JP |
| 3 | T Amin | International Journal of Food and Fermention Technology | IJFFT | Sher-e-Kashmir University of Agricultural Sciences & Technology, Kashmir | SKUAST, Kashmir |
| 4 | N Mahajan | Journal of Dairying Foods & Home Science | JDFSH | Punjab Agricultural University, Ludhiana | PAU, PB |
| 5 | B Yadav | Journal of Nursing Research, Education and Management | JNEM | Sri Guru Harkrishan Sahib College of Nursing, Punjab | SGHS CON, PB |
| 6 | D R Rao | European Journal of Clinical Nutrition | EJCN | National Institute of Nutrition (ICMR), Hydrabad | ICMR-NIN, HYD |
| 7 | N Joseph | Journal of Clinical and Diagnostic Research | JCDR | Kasturba Medical College, Manipal University, Mangalore | KMC, MAQ |
| 8 | Veena V | National Journal of Community Medicine | NJCM | BGS Global Institute of Medical Sciences, Bangalore | BGS GIMS, BLR |
| 9 | P V Kotecha | Journal of Health, Population and Nutrition | JHPN | Academy for Educational Development, New Delhi | AED, DL |
| 10 | V Sharma | IOSR Journal of Nursing and health Science | IOSR- JNHS | Baba Farid University Of Health Science, Punjab | BFUHS, PB |
| 11 | G Fathima | International Journal of Advanced Research in Science, Communication and Technology | IJARSCT | Ch. S. D. St. Theresa’s College for Women, Andrapradesh | CHSDSTTC, AP |
| 12 | V C Punitha | Journal of Pharmacy & Bio allied Sciences | JPBS | Meenakshi Academy of Higher Education, Chennai | MAHER, CN |
| 13 | D Chandar | International Journal of Adolescent Medicine and Health | IJAMH | Jawaharlal Institute of Postgraduate Medical Education and Research, Puducherry | JIPMER, PY |
| 14 | S Goel | International Research Journal of Biological Sciences | IRJBS | Kurukshetra University, Kurukshetra, Haryana | KUK, HR |
| 15 | M U Ramchandra | International Journal of Science and Research | IJSR | Krishna Institute of Nursing Sciences Karad, Maharashtra | KIMSDU, MH |
| 16 | A Gupta | Indian Journal of Public Health | IJPH | All India Institute of Medical Sciences, New Delhi | AIIMS, DL |
| 17 | Shanthini U S | IP Journal of Paediatrics and Nursing Science | IP-IJPNS | kaher Institute of Nursing Sciences, Karnataka | KINS, KA |
| 18 | C Bhushan | Centre for Science and Environment | CSE | Centre for Science and Environment, New Delhi | CSE, DL |
| 19 | G Shanthi | International Journal of Applied Research | IJAR | Sree Narayana Nursing College, Andra pradesh | SNN Col., AP |
| 20 | S Borgis | EPRA International Journal of Multidisciplinary Research | EPRA IJMR | University of Agricultural Sciences, Karnataka | UAS, KA |
| 21 | R Shukla | International Journal of Multidisciplinary Research and Analysis | IJMRA | Baba Saheb Bhimrao Ambedkar University, Lucknow | BBAU, LKO |
| 22 | N Vikraman | International Journal of Trend in Scientific Research and Development | IJTSRD | St. Teresa’s college, Ernakulam, Kerala | St. T. Col., KL |
| 23 | N Varma | Paripex- Indian Journal of Research | PIJR | Sarojini Naidu Govt. Girls Post Graduate College, Bhopal | SNGGPG, BHO |
| 24 | B A Ayushree | International Journal of Agro Nutrifood Practices | IJANP | Dr. Rajendra Prasad Central Agricultural University, Bihar | RPCAU, BR |
| 25 | N Haokip | Food Science Research Journal | FSRJ | Punjab Agricultural University, Ludhiana, Punjab | PAU, PB |
| 26 | J Oinam | International Journal of Scientific Research | IJSR | Regional Institute of Medical Sciences, Imphal | RIMS, IMF |
| 27 | R K Nayak | International Journal of Community Medicine and Public Health | IJCMPH | SDM College of Medical Sciences and Hospital, Karnataka | SDM Col., KA |
| 28 | T Khongrangjem | Clinical Epidemiology and Global Health | CEGH | Manipal University, Manipal | Manipal UnI., Manipal |
| 29 | M Meena | International Journal of Innovative Research and Review | JIRR | University of Rajasthan Jaipur, Rajastan | Univraj, JP |
| 30 | R Lalnunthara | Mizoram University Journal of Humanities & Social Science | MZUJHSS | Higher and Technical Institute Mizoram, Lunglei, Mizoram | HATIM, MZ |
| 31 | V Agarwal | Think India Journal | Think India J. | Jaipur National University, Jaipur | JNU, JP |
| 32 | D Naveenkumar | Adalya Journal | Adalya J. | Periyar University Salem, Tamil Nadu | PUS, TN |
| 33 | I Kuila | International Journal of Health Science and Research | IJHSR | Sarada Ma Girls College, Kolkata | SMGC, KOL |
